# The network approach to laboratory procurement and supply chain management: Addressing the system issues to enhance HIV viral load scale-up

**DOI:** 10.4102/ajlm.v9i1.1022

**Published:** 2020-08-13

**Authors:** Jason Williams, Dianna Edgil, Matthew Wattleworth, Clement Ndongmo, Joel Kuritsky

**Affiliations:** 1 Supply Chain Division, United States Agency for International Development (USAID), Crystal City, Virginia, United States; 2 Global Health Supply Chain Program, Procurement and Supply Management (GHSC-PSM), Arlington, Virginia, United States

**Keywords:** laboratory networks, molecular scale-up, optimisation, supply chain, laboratory

## Abstract

Investment in viral load scale-up in order to control the HIV epidemic and meet the Joint United Nations Programme on HIV and AIDS (UNAIDS) ‘90-90-90’ goals has prompted the President’s Emergency Plan for AIDS Relief and countries to increase their investment in viral load and infant virological testing. This has resulted in the increased procurement of molecular-based instruments, with many countries having challenges to effectively procure and place these products. In response to these challenges, the global laboratory stakeholder community has developed an informed ‘network approach’ to guide placement strategies. This article defines and describes the ‘network approach’ for laboratory procurement and supply chain management to assist countries in developing a strategic instrument procurement and placement strategy. The four key pillars of the approach should be performed in a stepwise fashion, with regular reviews. The approach is comprised of (1) laboratory network optimisation, (2) forecasting and supply planning, (3) the development of effective procurement and strategic sourcing to develop ‘all-inclusive’ contracts that provide transparent pricing, and the establishment of clear service and maintenance expectations and key performance indicators and (4) performance management to increase communication and planning, and promote issue resolution. Investments in the network approach will enable countries to strengthen laboratory systems and ready them for future laboratory needs. These disease-agnostic networks will be poised to improve overall national disease surveillance and assist countries in responding to disease outbreaks and other chronic diseases.

## Background

Of the 36.9 million people living with HIV, approximately half (21.7 million) are on antiretroviral therapy, and of those, four out of five are virally suppressed.^1^ Ensuring patients are on the most effective treatments relies on the availability and use of viral load (VL) testing. For this to be successful, clinicians must order the test, samples must be transported to the laboratory and results must be returned. The achievement of the third ‘90’ of the ‘90-90-90’ strategy of the Joint United Nations Programme on HIV and AIDS (UNAIDS), to ensure that 90% of patients that are on HIV treatment are virally suppressed, depends on the scale-up of laboratory capacity with an effective sample transport network and an efficient laboratory–clinic interface that facilitates responses to patient management issues related to adherence and treatment failure.

In 2013, the World Health Organization included VL monitoring in its treatment guidelines, with subsequent guidance and a recommendation for its use in 2014 and 2015.^2,3,4^ The addition of VL testing as the cornerstone of the UNAIDS, ‘90-90-90’ strategy has resulted in an investment in VL testing globally.^5^ Investments have been made to assist ministries of health in revising treatment policies, building laboratory capacity, and training and sensitising clinicians and patients on testing. To facilitate scale-up, there has been an effort to increase efficiencies by promoting procurement coordination between donors, to improve transparent pricing for reagents, and to implement procurement principles to address service and maintenance. The goal for coordination is to create a network of diagnostic capability that is nested within a broader public health response towards laboratory development.

As countries have attempted to take VL testing to scale, reoccurring challenges continue to surface, which include difficulty with procurement and sample transport, delays in the return of results and the need to increase clinical demand.^6,7,8^ These challenges have an impact on the ability to increase testing and ensure quality services. In order to address this, we promote a ‘network approach’. By this we mean the use of a systematic strategy that aligns capacity with utilisation, promotes efficiency in the procurement and placement of machines, enables collaboration between donors and countries, and focuses on the development of efficient sample transport and result return. The purpose of this article is to identify the key aspects of this approach and provide critical considerations for countries to improve performance and create network efficiencies in order to reach their diagnostic goals.

## Excess laboratory capacity

In most countries, instrument capacity is higher than needed, requiring significant growth in testing in order for these products to be optimally used. Even with a phased approach to the scale-up of VL testing, as recommended by the World Health Organization,^3^ only a portion of coverage goals have been achieved. In Zimbabwe, for example, the national VL testing coverage target was established at 21% in 2015, but only 5.6% coverage was achieved by year’s end, largely due to challenges with resource mobilisation and coordination, equipment procurement and specimen transport.^9^ By June 2016, of seven countries surveyed, four (Tanzania, Côte d’Ivoire, Malawi and Uganda) were performing less than 25% of the necessary VL tests needed for patients on antiretroviral therapy.^10^ In 2016, Médecins Sans Frontières (MSF) estimated the coverage of VL monitoring across seven sub-Saharan African countries to be variable, ranging between 32% and 91%.^6^ Data on infant virological testing (IVT) show less than 50% coverage within the first six weeks of life in many sub-Saharan African countries.^7^

**FIGURE 1:**
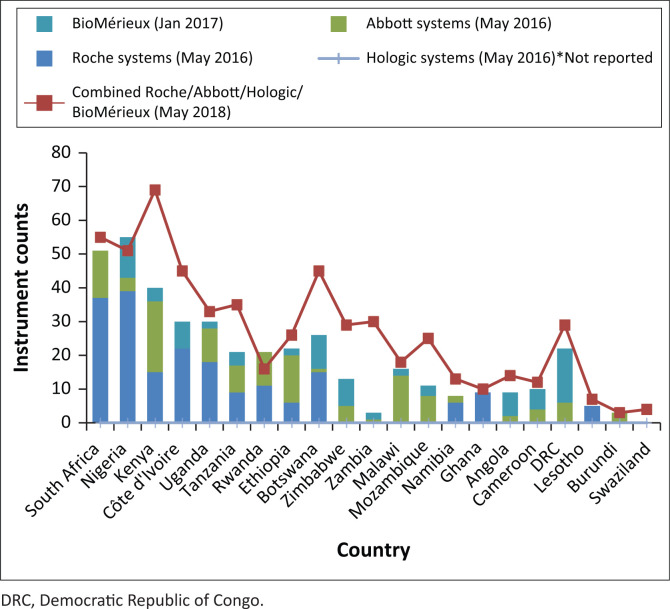
Reported manufacturer instrument counts in sub-Saharan Africa (May 2016 to May 2018, an increase from 405 to 569 instruments).

**FIGURE 2:**
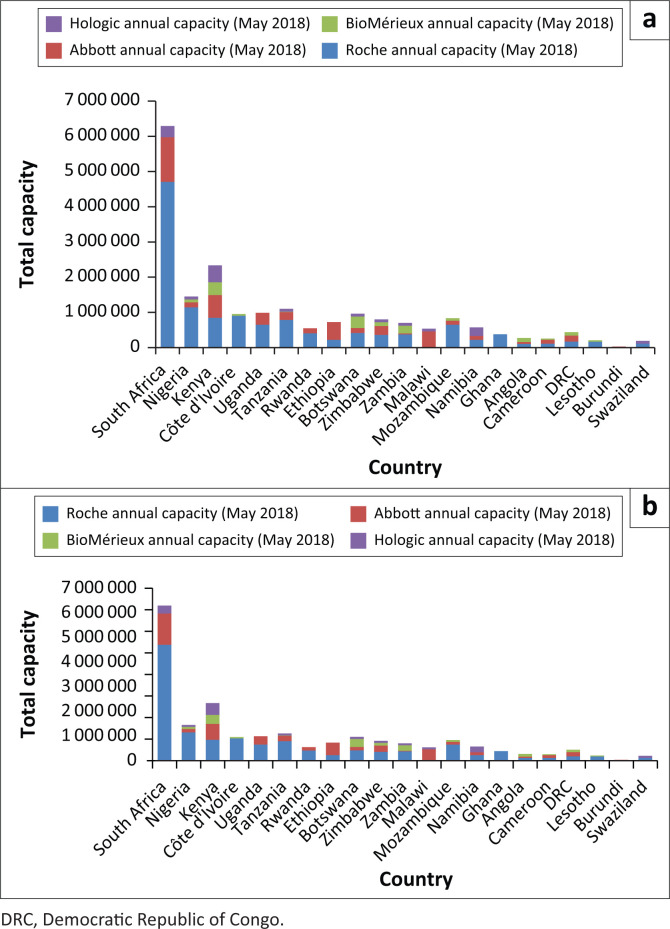
Diagnostic capacity estimates in sub-Saharan Africa (May 2018 – 20 580 136 tests).

The World Health Organization’s survey of data on diagnostic instruments from 2013, which assessed the scale-up of VL testing in 127 countries, showed that VL testing capacity was available to conduct 1.2 tests per person on antiretroviral therapy, but only 0.5 tests per person were conducted. This results in a capacity utilisation rate of only 36.5%.^11^ More recently, reports from major molecular instrument manufacturers demonstrate that countries continue to increase the number of instruments. Between May 2016 and May 2018, testing capacity in 21 African countries increased from over 15 million to 20.5 million tests, with an increase of 164 large molecular instruments (manufacturer reported, see Figure 1 and Figure 2). In most countries, existing instrument numbers and capacity are not limiting factors associated with the scale-up of VL testing.

Past scale-up efforts for CD4 testing resulted in uncoordinated procurement and underutilisation of instruments, suboptimal network expansion and a lack of service maintenance coverage across laboratory networks.^11,12^ As VL testing replaces CD4 in most sub-Saharan countries to monitor antiretroviral therapy, many of these issues are again resurfacing, including uncoordinated instrument management strategies.^6,13^ Lessons learned from the implementation of CD4 testing indicate the need for a more efficient model of procurement and service provision for VL and IVT programmes.

## Challenges with the scale-up of viral load testing in sub-Saharan Africa

We have identified four challenges that programmes must address in order to take VL testing to scale: (1) donor and stakeholder coordination and transparent pricing, (2) inconsistency of reagent availability (forecasting and supply planning), (3) ensuring functional instrumentation and (4) suboptimal laboratory network planning and sample transport strategies.

### Challenge 1 – Donor and stakeholder coordination and transparent pricing

Coordination between partners and governments to ensure the distribution of resources according to programme needs has been challenging, frequently resulting in the over-procurement or under-procurement of instruments and reagents that do not meet the testing needs of programmes.^14,15^

One core aspect of the alignment of effective global procurement is to create transparency in pricing, leveraging volumes and donor investments as part of negotiating influence. Pricing variability across countries has been described as a limiting factor to scale-up,^16^ creating challenges with budgeting. Many countries with budget limitations have historically paid more per test due to lower testing volumes, with more difficult infrastructural challenges to overcome as part of service delivery. Without coordination, donors can inadvertently undermine the ability to negotiate cost-effective testing strategies, with an end result of diminishing testing pools across instrument types, limiting negotiating influence and undermining volume pricing for tests performed nationally.

To clearly understand pricing, it is important to unpack costs for fair comparisons. For example, per test costs could be calculated based on the primary reagent only, or may include reagents, consumables, shipping and distribution. Pricing depends on volumes, instrument type, sample type, local versus international procurement, mode of import, inclusive service, maintenance costs, logistics costs, vendor management of reagent inventories and reagent rental or leasing arrangements. All of these components influence pricing for comparative purposes.

The Global Fund (GF) has negotiated global access pricing for low and middle-income countries. The two most commonly used molecular brands for VL testing and IVT are Roche Molecular Diagnostics and Abbott Molecular Inc. Commodity-related prices are set at a rate of $9.40 per test for Roche, which includes reagents and consumables, with ex-works terms (goods are available at the seller’s or manufacturer’s site and must be transported by the buyer), whereas Abbott’s pricing is based on volumes and duration commitments.^17^ The Abbott’s approach has resulted in pricing variability across countries of between $10.50 and $22.50 per test for core reagents, with an additional $2.50 for the necessary calibrators, controls and added consumables. This brings Abbott’s pricing to between $13.50 and $23.60 per test. Yet, based on volumes and multi-year commitments as well as national negotiating influence, some countries have further reduced these prices.

It should be noted that pricing schemes offered by Roche can also have country-specific variability due to ‘free carrier’ pricing (where the seller arranges and pays for shipping to the country of export) included in the reagent pricing, with shipping details not separately itemised. This creates challenges during budgetary planning sessions, since it becomes difficult to predict shipping costs and ensure that the global ex-works $9.40 reagent and consumable pricing is adhered to. Transparency in total cost breakdown is needed, as there is a perception that the pricing offered is different from the GF-published $9.40 per test pricing.

### Challenge 2 – Inconsistent reagent availability (forecasting and supply planning)

Reagent availability has been highlighted as one core obstacle to the scale-up of VL testing.^6,7,16^ Although reagent availability is a critical aspect of ensuring VL testing, stock-outs are a symptom of broader supply chain system issues and data flow challenges that have a negative impact on scale-up.

### Challenge 3 – Instrument functionality due to inadequate or absent service and maintenance

Ensuring adequate service and maintenance, warranty and preventive maintenance coverage for equipment is a significant challenge. To date, instrument and vendor oversight has been managed on an instrument-by-instrument basis, with limited coordination of management strategies, sometimes independently by stakeholders in the same country. This has resulted in multiple, separate contracts for individual instruments, often negotiated on different timelines, using non-standardised terms and with limited consistency in contract oversight and management.

Key performance indicators and reporting requirements that can be used to monitor vendor performance have not historically been included in contracts. This makes adhering to existing service contracts and the monitoring of vendor performance difficult, limiting both vendor accountability and the development of appropriate maintenance strategies.

### Challenge 4 – Weak laboratory and sample referral networks

Given the ever-changing laboratory network environment, sample transport and referral networks have grown organically, and do not necessarily reflect an efficient network approach. These networks quickly become outdated and require adjustments to not only reflect national needs (e.g. the addition of other diagnostics demands, point-of-care, the integration of new specimens, backup sample transport in the event of equipment breakdown), but also to look for efficiencies, and possible integration. Ultimately, the effects of a fragmented sample referral network can be far-reaching, ranging from increased operational costs across the entire network to improperly placed instruments and limited instrument utilisation.

## Solutions: Procurement and supply chain management

**FIGURE 3:**
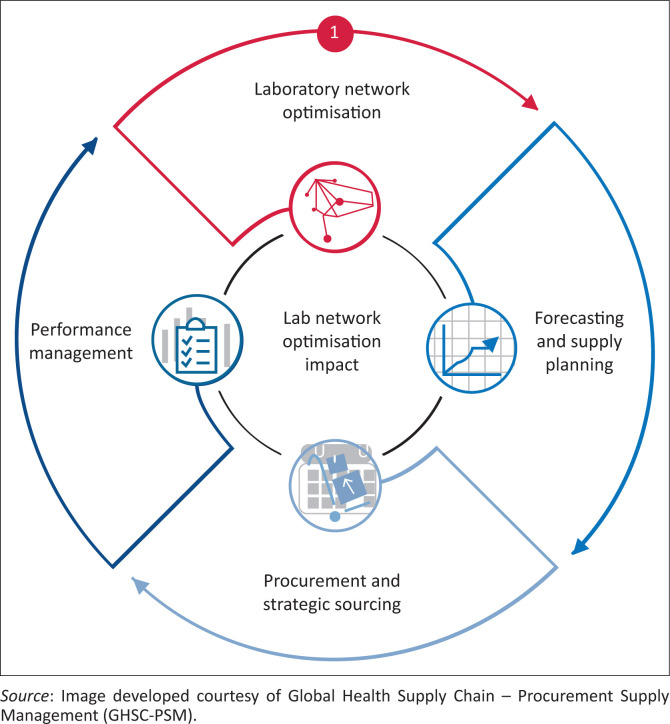
The network approach to laboratory procurement and supply management.

To effectively address these existing challenges, a holistic approach or network approach is needed, which spans four major building blocks or elements that are described below and summarised in Figure 3.

### Diagnostic network optimisation

The concept behind a network approach is to shift to an all-inclusive reagent rental scheme (RRS) or reagent service scheme (RSS) that serves all existing and new instruments. This approach would be national, and not specific to a stakeholder, donor or disease. A vendor-specific instrument contract would contain terms and conditions that are informed collectively by all stakeholders. This approach would require all stakeholders to take stock of existing instruments, service contracts and procurement pricing schemes, and establish jointly renegotiated terms that take all stakeholder investments and contributions to the overall network into consideration. Revised pricing schemes could potentially include:

- A national cost and contract structure that allows for volume growth and instrument expansion within a complete network, irrespective of the disease type or programme area, and that can be accessed by all stakeholders.
- A cost structure translated into an ‘all-inclusive per test cost’ spread across all instruments of the same brands within the network to include:
  ■ Cost options as part of network expansion that would account for existing legacy instruments and the development of new contract models (e.g. leasing and rentals) that facilitate the introduction of new instruments under standardised pricing schemes
  ■ Inclusive service and maintenance
  ■ Data solutions that would include patient result transmission, as well as instrument and user performance
  ■ Network staff training and consistency
  ■ Additional technology support that could assist in site-level efficiencies (barcode use, sample processing and workflow evaluations)
  ■ Enhanced commodity management strategies to ensure reagent availability (to include vendor-managed inventory)

The goal of a network approach is to improve instrument utilisation by aligning capacity with demand:

- Introducing standardised national pricing schemes, irrespective of the procurement mechanism, thereby enabling continuous service contract coverage
- Providing opportunities to amortise instrument costs into reagent costs, in order to lower startup costs associated with scale-up
- Sharing and assigning the longer-term management and mitigation of risks associated with instrumentation onto manufacturers and local vendors
- Providing a no-cost option for instrument replacements due to high failure rates, capacity issues (upgrading) or even outdated technology.

A network approach focuses on developing a baseline understanding of the current national VL testing network, including capacity and equipment utilisation, then exploring more efficient network options. Once a refined network is adopted, planning and procurement must be coordinated among all stakeholders to avert the addition of more instrumentation that may not be included in the planned diagnostic network, and ensuring the constant supply of reagents and consumables. In support of coordinated planning, it is important to develop criteria for the placement of additional machines, including point-of-care or near to point-of-care platforms and higher throughput platforms which all stakeholders would be required to adhere to. Negotiated agreements should look to the bundling of services (including connectivity). Contractual requirements for data sharing (downtime, testing protocols, specimen types, etc.) will facilitate management of the network in real time and improve vendor accountability.

To advance a network approach, it is important to look beyond a lowest price per test and focus on the total cost of ownership; initial per test costs will likely be higher, but the longer-term strategy will benefit the network.

#### Evidence-based optimisation of laboratory network

Factors determining the success of VL and IVT testing programmes include laboratory infrastructure and instrumentation, logistics, specimen transport, clinical implementation, and monitoring and evaluation. While it is helpful to coordinate procurement and service maintenance under a network approach, a limited understanding of reagent consumption, testing demand, laboratory performance and human resource capacity can undermine the functionality of a network. In cases of network expansion or revision, it may be necessary to carry out an analysis toward the goal of optimising the network. An approach to laboratory network optimisation would focus on the use of geographical information systems mapping tools (e.g. Laboratory Efficiency and Quality Improvement Planning [LabEQIP] software and Supply Chain Guru™ – LLamasoft^18,19^), to map laboratory network parameters, including instrument locations and utilisation, testing demands, quality assurance, human resources, sample transport lanes, specimen types, demographic needs, costing components and partner performance data. LabEQIP is a software tool developed by United States Agency for International Development (USAID) and LLamasoft, which is managed by the Global Health Supply Chain – Procurement and Supply Management (GHSC-PSM). It is a geographical information, systems-based solution that can improve laboratory network efficiency and advance quality service delivery through data-driven optimisation and modelling. Virtual modelling, prior to instrument placement, or as part of formalising an overall shift in testing strategies, is a critical component in informing the approaches to laboratory network optimisation. LabEQIP has most often been used to strategically plan the design of laboratory networks, the placement of equipment, the planning of sample referrals and the improvement of instrument utilisation. LabEQIP and Supply Chain Guru™ have been used in Nigeria, Cameroon, Rwanda, Eswatini, Zimbabwe and Zambia with support from the President’s Emergency Plan for AIDS Relief (PEPFAR), GF, the Clinton Health Access Initiative (CHAI) and GHSC-PSM, to develop virtual strategies to integrate HIV-tuberculosis sample transport, reduce instrument footprints to improve operational costs, and virtually place instruments to determine the impact on laboratory testing demands and instrument capacity requirements. LabEQIP can also be used to inform the integration of point-of-care technologies, and to assist in prioritisation and instrument rebalancing due to overburdened or underburdened testing demands.

### Demand forecasting and supply planning

In the initial phase of scale-up, programmes often use demographic or target-based forecasts. A demographic forecast takes the number of patients who are on antiretroviral treatment and multiplies that number by the number of VL tests per patient; a target-based forecast does the same, but uses the national or programme annual treatment numbers. Both types of forecast invariably overestimate commodity demand, as they do not account for unreliable laboratory or logistics information systems and poor reporting, poor site-level stock management practices, uncoordinated instruments and instrument failure.^6^ Further, during a period of rapid scale-up, historical consumption and procurement are not reliable indicators of future consumption.

**FIGURE 4:**
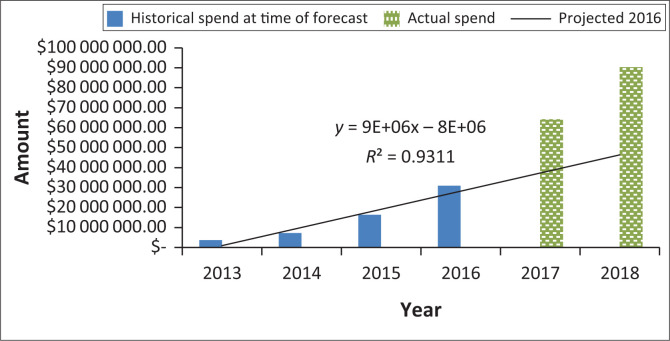
Linear projection of all the President’s Emergency Plan for AIDS Relief viral load testing demand based on 2013 to 2016 historical procurement (Projected 2016), compared to actual 2017/2018 procurement data.

USAID and PEPFAR, through its supply chain implementing partner, GHSC-PSM, has increased procurement of VL testing reagents from just over $7 million in 2014 to nearly $90 million in 2018. A linear projection of VL testing demand based on historical procurement in 2016 would have forecast approximately $37 million of VL testing-related procurement by 2017, increasing to $47 million in 2018. Actual 2017 VL testing procurement data reflected almost $6 million in GHSC-PSM expenditures, with over $90 million in procurement moving into 2018, an underestimation of about 44% and 48% if linear projections were used (Figure 4).

To address forecasting challenges, USAID promotes ForLab (forlabplus.com), which was developed by USAID and CHAI and is managed by GHSC-PSM, for national laboratory forecasting. ForLab has been used in more than 21 countries since its launch in 2013. ForLab includes forecasting commodity needs using a mixed methodology approach to improve accuracy and to provide consistent and greater transparency in national forecasting exercises. ForLab includes demographic and morbidity data, service statistics and logistics data on commodity consumption in an effort to triangulate multiple forecasting methods to derive a best-fit procurement plan that can be used by stakeholders to establish realistic budgets and supply planning activities.^19,20,21,22,23,24^ ForLab is a data-driven tool and works well when data are available and, when data are of a high quality, it can precisely predict need. However, poor site-level reporting can reduce its forecasting accuracy.

When highlighting stock-outs as a limiting factor associated with the scale-up of VL testing, it is important to acknowledge that there are many factors outside the supply chain that can impede improvements in reagent availability, which must be addressed concurrently. As programmes scale up, site-level storage space can become a challenge, causing the dispersal of reagents across various locations within a particular laboratory. Product dispersion can make routine stock management tasks more laborious and reduce reporting frequency and accuracy. As programmes scale up, it may be necessary to increase reagent distribution frequency to sites, if commodity storage is limited, for example from quarterly to monthly. For this to be successful, there is a need to ensure consistent and reliable stock status visibility. An additional factor not related to supply chains that has an impact on reagent availability includes early visibility into new instrument introductions, as without coordination additional reagents may not be available to support extra instrumentation.

In order to prevent stock-outs, programmes need effective data flow from testing sites to the central warehouse to guide inventory management practices and product distribution mechanisms. Effective supply chains are data-driven and require constant input on service delivery performance. Accurate and consistent commodity stock levels and consumption reporting improves supply chain systems, allowing for accurate forecasting, timely procurements and improved visibility for manufacturers to assist with manufacturing lead times for large order quantities. Without these consistent and reliable inputs through logistic management information systems or laboratory information management systems, it becomes increasingly difficult to prescribe effective procurement and supply chain interventions to reduce stock-out situations.

### Strategic procurement and sourcing

To address pricing variability within countries, it is critical to negotiate national pricing schemes. National testing volumes should be aggregated to negotiate a consistent price that all stakeholders can achieve. Donors, host-country counterparts and manufacturers can work collaboratively with aggregated volumes to derive transparent pricing schemes and mutually agreed upon prices that include additional service offerings outside of just reagents and consumables.

Recent coordination with the GF has resulted in price transparency in Haiti, the Democratic Republic of Congo and Cameroon, with initial price reductions of approximately $21.00 to $16.50, and then further to $13.50 for reagent costs. Efforts are still in process to promote further reductions as scale-up continues in these countries, as well as to include more comprehensive service packages. This includes service, maintenance and data management, as well as standardised reporting requirements informed by agreed upon key performance indicators as part of a price-per-test scheme.

The PEPFAR has currently renegotiated all of its existing VL/EID procurement contracts to significantly lower all-inclusive pricing schemes. A formal press-release will be announced shortly after the publication of this paper. The PEPFAR has engaged GF to push further transparent pricing reductions, with additional itemised system costing options, including all-inclusive reagent rental, service and maintenance, data management systems, as well as possible vendor-managed inventory.

**FIGURE 5:**
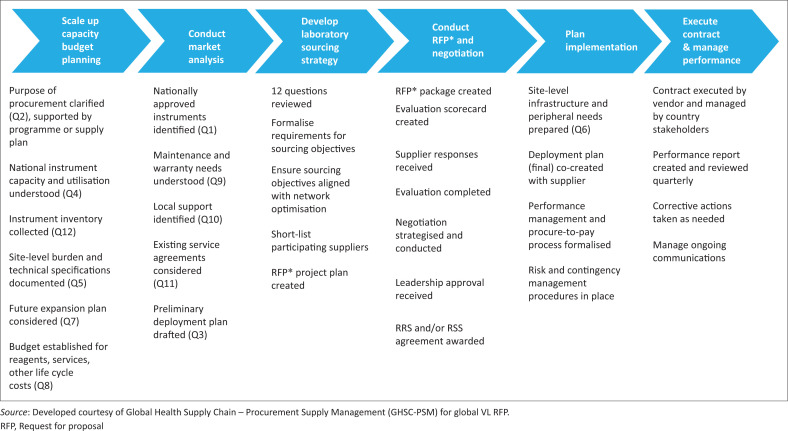
Approach for negotiating reagent rental agreements (linked to 12-question approach).

All future instrument investments and reagent procurement strategies should use RRS for new instruments, as well as inclusive RSS for existing instrumentation. The PEPFAR’s current country operational planning technical guidance emphasises the use of RRS for instrument expansion, driving countries towards a more systems-focused approach. Currently, South Africa, Kenya, Uganda^21,23^ and, more recently, Mozambique, Haiti and Nigeria are taking advantage of RRS or a combination of RRS and RSS. Currently, USAID is working with GHSC-PSM to introduce more dynamic RRS agreements in Nigeria, Mozambique, Haiti and Zambia. By moving to a RRS or a RSS approach, countries can amortise their initial capital investment for the scale-up and servicing of VL-testing instruments within their reagent pricing scheme, offsetting initial scale-up costs and expanding instrument coverage, as well as ensuring complete service contract availability. In order to assist countries in this approach, USAID developed a ‘12 question’ approach designed to help countries think through the use, placement and servicing of laboratory instruments prior to initiating procurement or RRS or RSS arrangements (Box 1, Figure 5).^18^

BOX 1 The ‘12 question’ approach to instrument procurement and placement.

**Table:** 

1	Is the product on the nationally approved list, for example national registration, national standardisation list?	Ensuring proposed instruments are approved for use in-country eliminates delays with instrument and commodity deliveries and reduces instrument and commodity diversity. Having fewer products flowing through the supply chain enhances the agility, efficiency and manageability of national laboratory supply chains.
2	Is the request to replace existing, old instruments? If yes, is there an instrument replacement strategy?	If the request is to replace existing instruments with a new brand of instrument, national supply plans and commodity availability will be impacted. Ensuring that an instrument replacement strategy is in place will assist in making reagent supply planning and procurement adjustments. If the replacement is for a similar instrument, these adjustments may not be necessary.
3	If these instruments are for new locations, is there an instrument deployment plan for the proposed instrument?	If the request is to add additional instruments, national supply plans and commodity availability will be impacted. Ensuring that an instrument deployment strategy is in place will assist in making reagent supply planning and procurement adjustments. Ensuring that the deployment plan delineates when each recipient laboratory is intended to come online will improve commodity planning for larger instrument procurements. Purchasing all instruments and all planned reagent requirements at one time can lead to significant reagent expiries if there are delays in instrument deployments. Active monitoring of the deployment plan can eliminate these issues.
4	What is the current estimated diagnostic capacity for this particular instrument type in the country?	Understanding current national instrument capacity will determine whether adding additional capacity could potentially be unnecessary. Network mapping could assist in ensuring appropriate placement if access is potentially informing the decision to add additional capacity even though aggregated capacity significantly exceeds testing demands.
5	What is the diagnostic burden at the proposed sites? Is the instrument selected appropriate, based on instrument capacity vs diagnostic demand?	Establishing the testing demand at the proposed instrument location will ensure that an appropriate instrument is selected based on the instrument capacity and testing needs. Procuring too large an instrument will lead to poor utilisation and an unnecessary cost burden for managing national laboratory networks; alternatively, procuring a low-volume instrument may require replacement sooner.
6	Is there suitable infrastructure at the proposed sites – are there any additional peripheral needs?	It is critical to determine site readiness as part of the deployment strategy development. If there are infrastructure needs, have they been budgeted for, and have arrangements been made to procure additional ancillary equipment or infrastructure renovations? Any delays will have an impact on the installation and clinical use, and could lead to reagent expiries if the deployment strategy is not actively monitored.
7	Is there expected service delivery expansion at the proposed sites? (scale-up)	As a follow-up to question 5, does the selected instrument account for a known increase in service provision or programme growth? Again, procuring too large an instrument will lead to poor utilisation; alternatively, procuring a low-volume instrument may require replacement sooner.
8	Have the additional costs of reagents, staff training and maintenance been considered – what are the funding sources and estimated costs?	Adding a replacement instrument of the same brand or type at an existing laboratory will not have an impact on reagent planning and training, but adding a new brand of instruments will require a new line of reagents, staff training and new maintenance arrangements. Understanding these costs, gaps and identifying who will cover them is critically important as part of planning prior to initiating procurements.
9	Will the instrument require an extended warranty after its warranty expires?	Making plans to coordinate extended warranties and initiating arrangements for service and maintenance after warranties expire will eliminate gaps in service coverage. Many instruments include initial warranties but after they expire, a lack of dedicated funds and forward planning often leaves instruments without active service contracts.
10	Is a local authorised manufacturer/distributor available to service the instrument?	If a local authorised distributor is not available in-country, delays in servicing can be expected. As part of the procurement and service contract negotiations, requests can be made to ensure a service representative as well as spare parts are available in-country.
11	Is there a maintenance service agreement (MSA) in place for similar instruments you have currently on hand? If yes, is the MSA still valid and who is managing the agreement?	If there are similar instruments already in-country which are covered under an existing MSA, can these additional instruments be added? If there are similar instruments that are not covered under an active service contract, it is important to include these instruments to ensure reduced instrument downtime, and to possibly negotiate a network MSA covering all instruments, over an individual instrument MSA. This approach can assist in reducing MSA costs and promote efforts to develop a national MSA strategy with a harmonised set of key performance indicators.
12	Is an equipment inventory list available for similar instruments on hand? If so, was an inventory conducted in the past 12 months with updated serial numbers and site locations?	Having a national equipment inventory on hand can guide negotiations for a national MSA and can also assist in mapping all instruments as part of developing a broader national network approach. Having a complete national inventory of instruments can also assist with planning for the design of the overall national laboratory network, instrument placement and sample referral strategies.

*Source*: World Health Organization. Guidance for procurement of in vitro diagnostics and related laboratory items and equipment [homepage on the Internet]. 2nd ed. 2017. ISBM 978 92 4 154866 3. [cited 21 Mar 2019]. Available from: https://apps.who.int/iris/bitstream/10665/255577/1/9789241512558-eng.pdf

### Monitoring instrument and vendor performance

When considering RRS or RSS contracts, it is critical to establish defined expectations. Contracts should be negotiated collaboratively with all stakeholders and donors. A harmonised set of key performance indicators (Table 1) should be developed and should include: minimum response times for instrument repairs, training, logistics, and instrument and end-user performance. Clear thresholds should be established for instrument failure frequencies, and service providers should be held accountable for responding to site-level failures that go beyond these established thresholds. Contracts should dictate a standardised monthly and quarterly reporting format to assist in addressing site or instrument-specific challenges, as well as vendor service delivery issues. The contract should also define at least quarterly meetings with the supplier to review performance and work collaboratively to solve problems and address any performance issues. Contracts should also clearly delineate lists of parts to be made available in-country for high-failure parts, minimum service technician requirements, possible data solutions for patient result transmission, and monitoring instrument and end-user performance.

**TABLE 1:** Illustrative key performance indicators used to monitor vendor service and instrument performance through service contracts.

No.	Key performance indicator	Unit of measure	Definitions	Frequency of data collection	Target (negotiated with the vendor)	Applicable service type
1	Percentage of preventative maintenance visits performed on schedule as per the terms of the subcontract	Percentage	Number of visits made on schedule/total number of visits as per the subcontract. ‘On schedule’ is defined as the date (±3 calendar days) listed as per the terms of the subcontract	Monthly	100%	Preventative maintenance
2	Number of calendar days lapsed from operator’s initial service call to service provider’s response	Calendar days	Day of call by the operator = Day 0. Each day lapsed after call by the operator and before service response rendered = +1. A response is not the same as service completion. It means that there was either an attempt to fix the machine (remotely or on-site) or an attempt was made to collect more information in order to diagnose the problem. A response to the call is not necessarily a solution	Monthly	Within 24 hours. Each incident must receive a visit within 24 hours	Repair
3	Number of calendar days lapsed from equipment diagnosis to the arrival of the spare parts at the site	Calendar days	Day of equipment diagnosis = Day 0. Each day lapsed before the arrival of the spare parts at the site = +1	Monthly	≤ 3 business days for minor repairs; ≤ 10 business days for major repairs	Preventative maintenance and repair
4	Number of calendar days lapsed from initial service call to job completion	Calendar days	Day of call by the operator = Day 0. Each day lapsed that the equipment is not fully functional = +1. Job completion = date of signed job card by the operator, which is an indication that the machine is fully operational	Monthly	≤ 3 business days for minor repairs; ≤ 10 business days for major repairs	Repair
5	Number of analyser outages that occur less than 3 months after any scheduled or unscheduled maintenance or repair work	Number of analyser outages	An analyser outage is any malfunction that diminishes the efficiency, effectiveness or use of the equipment. The purpose is to capture any defects (per machine preferably) which seem to be recurring or are not completely addressed even after the job card has been signed	Monthly	< 2 per year	Preventative maintenance and repair
6	Percentage of service reports submitted on time as per the terms of the subcontract	Percentage	Each service report must include a complete schedule and status update on maintenance or repair for all equipment: a functional status summary of all equipment currently under contract, job cards and resolution notice for any service or maintenance performed within the reported period and KPI status report, which includes performance targets, actual performance metrics for the period and a cumulative total	Cumulative basis for an annual average	100%	Preventative maintenance and repair
7	Percentage of rejected runs or failed tests	Percentage	Number of valid test results/number of test runs. ‘Run’ is defined as loading prepared samples into the selected platform for analysis	Monthly	No greater than 5%	Failure rates greater than 5% require the vendor to conduct a root cause analysis and submit a formal report. This report should include the reason for the failure rates, a replacement plan for failed reagents, and a remedial action plan. This would include refresher training, the replacement of reagents and instrument upgrades as needed

*Source:* Developed courtesy of Global Health Supply Chain – Procurement Supply Management (GHSC-PSM)

KPI, key performance indicators.

### Conclusion

The current effort to scale up VL testing and IVT has been significant. Gains have been achieved within national laboratory networks to scale up VL testing and IVT, but there is still a need to ensure sound investments in laboratory infrastructure and instrumentation, without overlooking the supportive structures of logistics, clinical components, and monitoring and evaluation protocols. There are lessons learned from past scale-up efforts for CD4 testing, with the current global strategy to ensure procurement coordination across donors, standardising and ensuring transparent pricing for reagents, and implementing general procurement principles that aim to address some of the main supply chain and service challenges. However, these global strategies must be translated into operational plans at a country level. To be successful, all stakeholders will need to embrace the full cycle of the network approach for laboratory procurement and supply chain management; take stock of existing instruments, service contracts and procurement pricing schemes; and establish jointly renegotiated terms that leverage all stakeholder investments. Countries that have successfully scaled up VL testing and IVT have focused on making these commitments and have thereby reduced the risk of equipment failure and commodity stock-outs - two critical challenges to the success of VL testing and IVT programmes.

While each of the four pillars of the network approach for procurement and supply management can support elements of the supply chain, true transformation of the laboratory network is only possible through embracing all four of the strategic pillars in a stepwise approach, with each phase in the cycle continuing to inform the next step.

In the longer term, these investments and the broader network approach will not only address some of the more immediate challenges, but will also enable countries to strengthen laboratory systems and ready themselves for implementing future laboratory needs. These disease-agnostic molecular networks will be poised to improve overall national disease surveillance and assist countries in responding to disease outbreak responses and other chronic diseases. In addition, such networks will position countries to address sustainable strategies for laboratories in future health agendas.
